# High‐Sensitivity Detection of Urinary Extracellular Vesicles With Upconverting Nanoparticle‐Based Lateral Flow Immunoassay

**DOI:** 10.1002/jex2.70053

**Published:** 2025-07-06

**Authors:** Md. Khirul Islam, Imran Mahmud, Klinton Ali, Teppo Salminen, Pekka Taimen, Peter J. Boström, Janne Leivo, Urpo Lamminmäki, Iida Martiskainen

**Affiliations:** ^1^ Biotechnology Division, Department of Life Technologies and FICAN West Cancer Centre University of Turku Turku Finland; ^2^ InFLAMES Research Flagship University of Turku Turku Finland; ^3^ Uniogen Oy Turku Finland; ^4^ Institute of Biomedicine, University of Turku, and Department of Pathology Turku University Hospital Turku Finland; ^5^ Department of Urology Turku University Hospital and University of Turku Turku Finland

**Keywords:** bladder cancer, lateral flow assay, rapid test, tetraspanin CD63, upconversion luminescence, urinary EVs

## Abstract

Urinary extracellular vesicles (uEVs) are well‐known to express tetraspanin family membrane proteins abundantly on their surface. In this study, we aimed to develop an upconverting nanoparticle (UCNP)–based lateral flow immunoassay (UCNP‐LFIA) designed for the rapid and high‐sensitivity detection of CD63‐positive uEVs for direct urinalysis. The assay utilizes UCNPs reporter to detect low concentrations of EVs. Minimally processed uEV samples from bladder cancer (BlCa) (*n* = 62), benign prostatic hyperplasia (BPH) (*n* = 50) and healthy (*n* = 30) individuals were tested in sandwich UCNP‐LFIA format, capturing uEVs with the same anti‐CD63 antibody conjugated to UCNP and immobilized on the test zone. After 80 min, the strips were read with an upconversion luminescence reader device. This UCNP‐LFIA measured CD63‐positive EVs with high sensitivity, exhibiting a limit of detection (LoD) of 4 × 10^7^ EVs/mL. The concentration of CD63‐positive EVs in BlCa patients showed a 2.3‐fold increase compared to benign conditions (*p* = 0.007), and a 16‐fold increase compared to healthy controls (*p* = 0.00001). The results demonstrate the potential of UCNP‐LFIA platform for sensitive and quantitative detection of uEVs, highlighting its promise as a tool for EV detection at point‐of‐care diagnostics.

## 1 Introduction

The urinary extracellular vesicles (uEVs) are secreted from cells along the urinary tract and carry various molecular contents such as proteins, nucleic acids and lipids reflecting the state of their cells of origin (Smith et al. 2024). Tetraspanins, particularly CD63, are among the most widely studied putative biomarkers on uEVs, for their potential use in characterization and analysis (Welsh et al. 2024). We and others have utilized CD63 to develop simple immunoassays for uEV detection (Islam et al. 2019; Duijvesz et al. 2015; Cashikar and Hanson 2019). Several studies have reported that CD63‐positive uEVs are differentially released to the biofluids of the prostate (Duijvesz et al. 2015) and bladder cancer patients (Liang et al. 2017) compared to healthy individuals, highlighting their diagnostic potential. Moreover, elevated levels of CD63‐positive EVs from other body fluids have been observed in different cancer types such as pancreatic (Odaka et al. 2022), melanoma (Logozzi et al. 2009), breast (Terävä et al. 2022), renal (Khan et al. 2024) and gastric cancers (Miki et al. 2018).

Lateral flow immunoassay (LFIA) is a widely used assay concept with ideal features for use in decentralized settings, including a user‐friendly format, short assay time and the ability to offer instant results in resource‐constrained settings (Patil et al. 2022). This immunochromatographic method enables the detection and quantification of target analytes on a nitrocellulose‐based strip (Bayoumy et al. 2020). However, traditional LFIA using colloidal gold nanoparticle‐based labels often lacks sensitivity, making it inadequate for detecting low concentrations of analytes like uEVs from urine due to their dilute nature.

In this study, we utilized a highly sensitive upconverting luminescence nanoparticles (UCNPs)‐based LFIA concept to address the limitations of detecting uEVs. UCNPs are crystalline lanthanide‐doped nanoparticles excited with near‐infrared radiation (NIR), allowing quantitative biomarker detection in biological samples (Máčala et al. 2024). This and many other unique properties of UCNPs, such as stability, resistance to photobleaching and ability to eliminate background autofluorescence originating from the sample matrix or test device component through the anti‐Stokes phenomenon, make UCNPs a highly promising reporter system for high‐sensitivity immunoassay development (Martiskainen et al. 2021). UCNPs reporter technology has been shown to have beneficial analytical features in LFIA development, offering high sensitivity and resilience for complex sample matrices which are crucial features for rapid tests (Salminen et al. 2020).

In this study, we developed a highly sensitive UCNP‐LFIA to detect CD63‐positive uEVs directly from urine. Along with the high sensitivity for uEV detection, the CD63‐UCNP‐LFIA shows promise in distinguishing bladder cancer from benign and healthy individuals.

## 2 Materials and Methods

### 2.1 Reagents and Apparatus

The components of the lateral flow strips were nitrocellulose membrane: LFNC‐C‐BS023‐70 (Nupore membranes, Ghaziabad, India), sample pad: Ahlstrom 8951(Ahlstrom‐Munksjö, Finland) and wicking pad: C083 CFSP223000 cellulose fibre pad (Merck Millipore, Massachusetts, USA). Monoclonal antibodies targeting CD63 (clone 556019) were purchased from BD Bioscience (Vantaa, Finland). Rabbit Anti‐Mouse antibody (RAM) was obtained from Invitrogen (Massachusetts, USA). DBCO‐PEG4‐NHS was purchased from Merck (Sigma‐Aldrich). Luminescent UCNP reporters (RD Upcon540‐L‐C4) were obtained from Uniogen Oy, Finland. Buffer consisted of 0.05‐M Tris, pH 7.5, 0.300‐M NaCl, 0.04% NaN3, 1 x Upcon stabilizer, 1.5% BSA and 0.1% Tween‐20. In addition, Linomat 5 printer (Camag, Muttenz, Switzerland) was used for antibody dispensing on the nitrocellulose membrane. Biodot CM4000 Guillotine (BioDot Inc., California, USA) was used to cut the membranes into uniform strips.

### 2.2 Urine Samples Information

Urine samples were collected from healthy individuals as well as those diagnosed with bladder cancer (BlCa) and benign prostatic hyperplasia (BPH). The healthy urine samples were acquired from the Biotechnology Division at the University of Turku in 2018–2020, while those from individuals with bladder cancer and BPH were obtained from the Turku Prostate Cancer Consortium (TPCC), spanning the period of 2013–2018. This study utilized 30 healthy samples (males: 18, females: 12; median age: 33 years), 50 BPH samples (all males; median age: 72.9 years) and 62 bladder cancer samples (males: 57, females: 5; median age: 70.1 years). Urine samples from bladder cancer patients were collected by catheterization immediately prior to radical cystectomy or transurethral resection of bladder tumour (TURBT). Similarly, age‐matched urine samples from BPH patients were collected as benign controls before transurethral resection of prostate (TURP). No pre‐processing of urine, such as centrifugation or filtration, was performed prior to the storage of sample. However, urine samples were stored at −80°C until further use. Before using the sample into assay well, pre‐processing of urine was done by centrifugation at 2000 × *g* for 5 min +4°C to eliminate cells and debris. As a part of ethical statement, all participants provided their informed consent prior to inclusion in the study. The study was conducted in accordance with the regulations and guidelines of the Helsinki Declaration. The research protocol involving clinical materials was authorized by the Ethics Committees of the Hospital District of Southwest Finland and the University of Turku, Finland (statement number 56/2018 for healthy controls and ETMK Dnro: 3/1801/2013 for bladder cancer and BPH samples).

### 2.3 EV‐Stripped and EV‐Concentrated Sample Preparation

To produce EV‐stripped urine (EV removed sample), an ultrafiltration concentrator Vivaspin 2 column (polyether sulfone nanomembrane, MWCO, 100 kDa, Sartorius, Germany) was used. The column was pre‐washed with 1X PBS to remove glycerol and preservatives. Then, 10 mL of urine samples were passed through the column by centrifuging at 2000 × *g* for 10 min. The resultant flow‐through was collected and used as a blank for the assay. Since EVs typically range from 40 to 100 nm in size and the filtration membrane restricts particles larger than 10 nm, the flow‐through is expected to be free of EVs. At the same process, EVs‐concentrated (EVs‐enriched) urine was collected as concentrated samples by reversing the Vivaspin column using 2000 × *g* centrifugation for 2 min, yielding 1 mL of EV‐concentrated urine from the initial 10‐mL urine.

### 2.4 Cell Culture Medium and EV Isolation

We used EVs from the cell culture medium (CCM) of the DU145 prostate cancer cell line, which was purchased from ATCC (Teddington, UK). Cells were maintained in RPMI‐1640 medium and cultured at Integra bioreactor flask (Integra Biosciences Corp, Hudson, USA), supplemented with L‐glutamine (2 mM), penicillin (100 U/mL), streptomycin (100 µg/mL) and 10% foetal bovine serum (FBS) (Mitchell et al. 2008). EVs from FBS were depleted by ultracentrifugation (UC) at 100,000 × *g* for 18 h followed by serial filtration (0.22 µm followed by 0.1 µm) using vacufilter units (Millipore). The CCM was collected upon reaching the confluency 80%–100%. To remove cells and cell debris, the CCM was subjected to serial centrifugation (400 × *g* for 10 min followed by 2000 × *g* for 15 min). EVs from DU145‐CCM were isolated using UC by floating them on a 30% sucrose/D_2_0 cushion, followed by 100,000 × *g* for 75 min at 4°C using a SW‐28 rotator (Beckman Coulter, Fullerton, California, USA) and then washed with PBS (Lamparski et al. 2002; Théry et al. 2006). Finally, EVs were stored at −80°C until further use.

### 2.5 EV Characterization Methods: NTA, TEM and TRF Immunoassay

The characterization of EVs follows the guidelines set by MISEV2023 to ensure quality control and reproducibility in EV research (Welsh et al. 2024). Nanoparticle tracking analysis (NTA) was performed using a NanoSight LM10 system configured with temperature‐controlled LM14 laser module with a 488‐nm laser following the previously published article (Welton et al. 2016; Webber and Clayton 2013). For each sample, three 30‐s videos were recorded with a camera. Recorded videos were analysed with the concentration and size distribution of measured particles with corresponding standard error. Each NTA measurement was performed in triplicate, using default threshold parameters to minimize bias. For ideal measurements, samples were diluted with PBS so that the concentration was within 3 × 10^8^ − 1 × 10^9^ particle/mL.

Transmission electron microscopy (TEM) was conducted using a JEM‐1400 plus TEM (Jeol, Tokyo, Japan) operated at 80 kV, following the protocol detailed in a previously published article (Welton et al. 2016; Islam et al. 2022). The EVs were adsorbed onto formvar‐coated grids for 20 min and subsequently fixed in 1% (v/v) glutaraldehyde for 5 min. The grids were then washed three times with PBS and three times with distilled water. For contrast enhancement, the grids were negatively stained with a 2% methyl cellulose/uranyl acetate (0.4%) solution for 10 min. Finally, the grids were air‐dried before observation under TEM.

Time‐resolved fluorescence (TRF) immunoassay was performed to assess EVs surface marker display, as previously described (Islam et al. 2019). Briefly, biotinylated capture antibodies, including anti‐CD63 and anti‐CD81, were immobilized on 96‐well streptavidin‐coated microtiter plates. Following this, 500 ng of EVs per well were added in 30 µL of RED assay buffer (product #: 42‐02TY, Uniogen Oy, Finland). As a blank control, a fresh CCM was used. After 1‐h incubation at room temperature (RT) with gentle shaking, the wells were washed twice before adding 1 × 10^7^ of anti‐CD63‐ or anti‐CD81‐EuNPs in a final volume of 30 µL. The NPs were incubated for 1 h at RT with gentle shaking (40 × *g*). TRF (TRF europium at *λ*_ex_: 340 nm; *λ*_em_: 615 nm) was measured from the well surface using a Victor1420 multi‐label counter (PerkinElmer).

### 2.6 Preparation of the Reporter

Luminescent reporter UCNPs were obtained from Uniogen Oy, Finland. The utility and efficacy of UCNPs as luminescent reporters have been described previously (Raiko et al. 2021; Palo et al. 2018; Ekman et al. 2024). In this study, monoclonal anti‐CD63 antibody (0.2 mg) was conjugated to UCNPs (2 mg; RD Upcon540‐L‐C4, Uniogen Oy) according to the manufacturer's instructions. The conjugate was washed three times with washing buffer (20‐mM borate buffer, 150‐mM NaCl, 0.05% Tween 20) to remove unconjugated antibodies by pelleting the UCNPs using 17200 × *g* centrifugal force for 20 min at 4°C. The CD63‐UCNP conjugates were stored as a stock in a final buffer volume 200 µL at +4°C in storage buffer (20‐mM borate buffer, 150‐mM NaCl) with 1x Upcon Stabilizer (Uniogen Oy).

### 2.7 Assembly of the LFIA Strips

In the sandwich immunoassay, anti‐CD63 was used as a capture antibody that was dispensed on the test line of the nitrocellulose membrane at a distance of 10 mm from the start of nitrocellulose, using a concentration of 1 mg/mL (1000 ng/cm), into 10‐mM Tris HCl buffer at pH 8. The control line, consisting of RAM antibody with a concentration 0.6 mg/mL (600 ng/cm), was printed 5‐mm downstream from the test line using the same buffer. Both lines were printed by Linomat 5. After printing, the strips were dried overnight at +35°C. The sample and absorbent pads measured 10 and 30 mm, respectively. The sample pad was blocked by saturating it with a blocking buffer composed of 10‐mM borate buffer (pH 7.5), 0.05% Tween‐20 and 1% BSA, followed by drying for 2 h at +35°C. Then the sample pad and absorbent pad were laminated onto a single card with the nitrocellulose with a 2‐mm overlapping between the materials. Finally, the assembled cards were cut into 4.8‐mm wide strips using Biodot CM4000 Guillotine cutting system.

### 2.8 CD63‐UCNP‐LFIA Protocol

**FIGURE 1:**
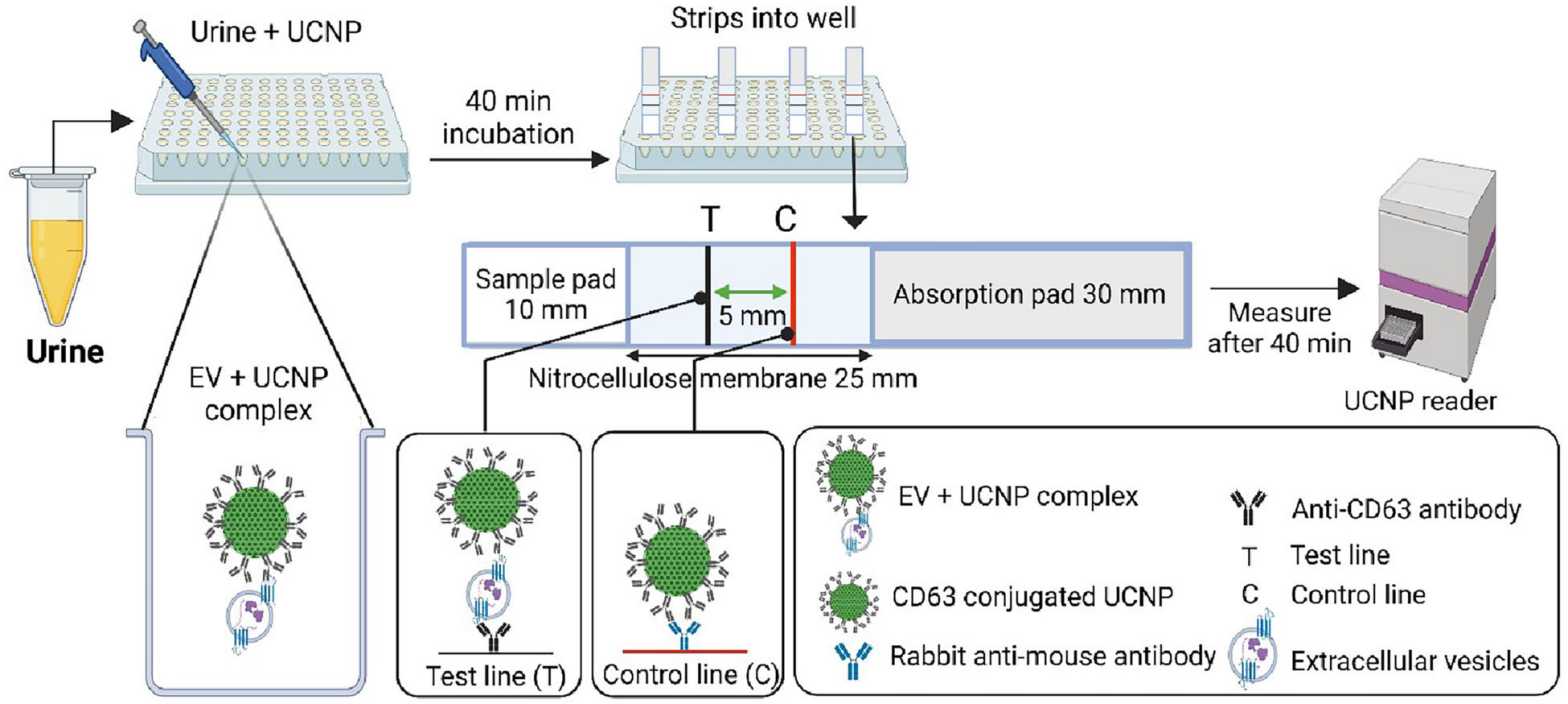
Schematic of the immunoassay for detection of CD63‐positive EVs using upconversion nanoparticles (UCNPs). LF‐strips were prepared with a sample pad, absorbent pad and nitrocellulose membrane. The nitrocellulose membrane contains a test line (T) with anti‐CD63 antibody and a control line (C) with Rabbit Anti‐Mouse antibody. During pre‐incubation (40 min), the EV‐UCNP complexes are formed and later captured and measured on the test line (T).

In this work, a quantitative LFIA was conducted using CD63‐conjugated UCNPs to detect CD63‐positive EVs. Four sources of EV samples were utilized as follows: EVs derived from the DU145 cancer cell line as a standard, as well as urinary EVs from healthy, benign and bladder cancer urine samples. Figure 1 depicts the setup of the immunoassay method for the CD63‐UCNP‐LFIA.

Urine samples from healthy, benign and bladder cancer patients (40 µL/well) were added into microtiter plate wells. A 7.5% BSA in TSA and EV‐stripped urine matrix were used as a negative control. Following the addition of the samples, UCNP dilutions were added into the same wells (25 µL/well) and incubated for 40 min with slow shaking. Then LF‐strips were placed into wells until the liquid was completely absorbed into the strip, typically taking 3–4 min. Then, the LF‐strips were transferred to separate wells containing 35 µL of assay buffer for washing and allowed to sit for 40 min. Finally, the strips were measured with the Upcon reader, which detected luminescence at 540 nm upon excitation of the UCNPs at a wavelength of 976 nm.

### 2.9 Sensitivity Determination of Assay

**FIGURE 2:**
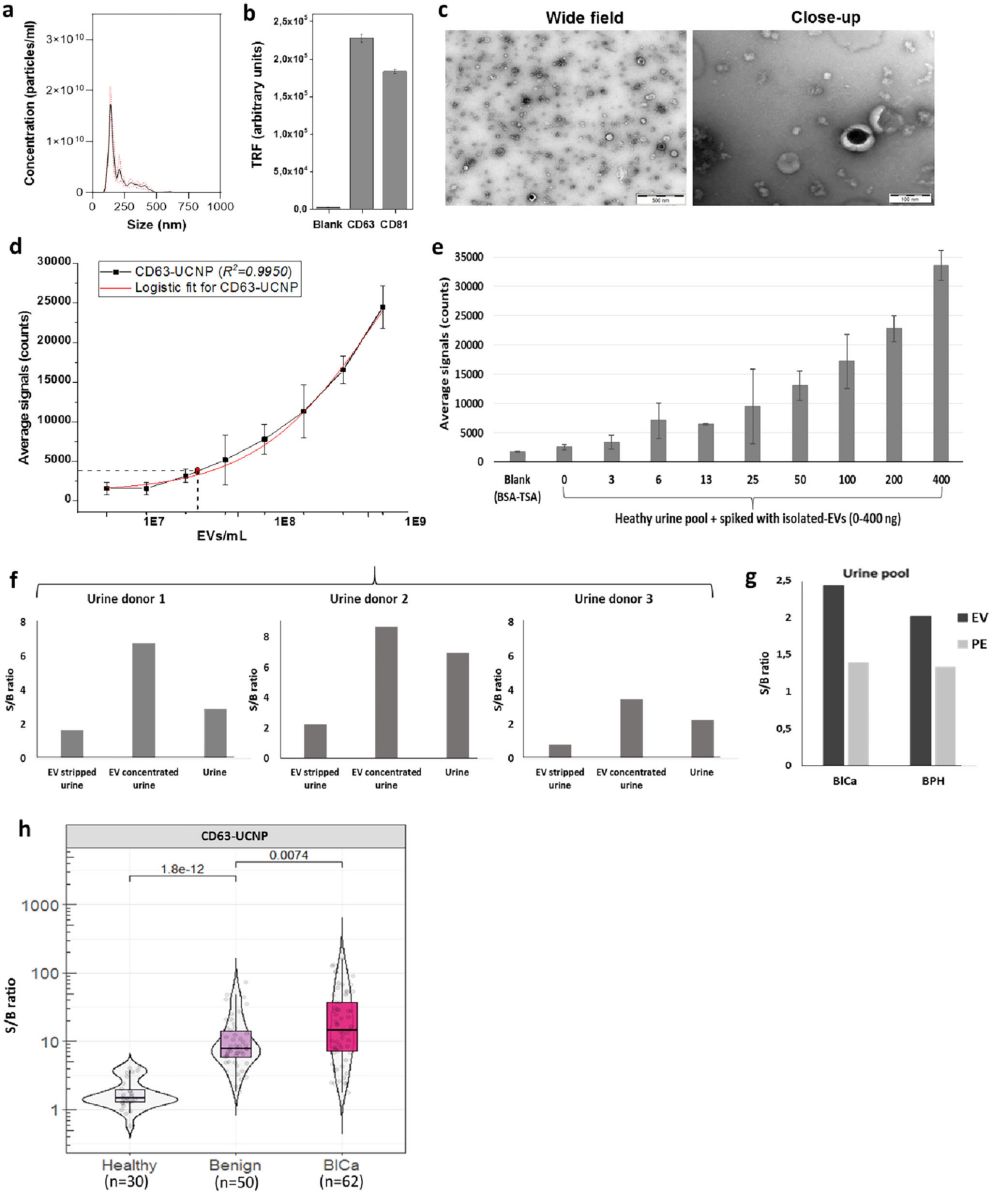
Characterization of DU145‐EVs and evaluation of CD63‐UCNP‐LFIA performance in DU145‐EVs and urine samples. (a) NTA of DU145‐EV shows the size distribution of particles. (b) TRF immunoassay indicates tetraspanin CD63 and CD81 markers expression on DU145‐EV. (c) TEM images indicate the shape and size of DU145‐EV at low magnification (left panel, scale bars: 500 nm) and their characteristic lipid bilayer structure at high magnification (right panel, scale bars: 100 nm). (d) Sensitivity of CD63‐UCNP assay using DU145‐EV, where the black line represents assay data for LoD determination, and the red line shows a logistic fit. (e) Matrix effect of CD63‐UCNP assay, where the response curve shows a linear increase of signal with increasing concentration of DU145‐EV spiked into a healthy urine pool matrix. Here, the error bars show standard deviations of three replicate LFIA strips. (f) Specificity of CD63‐UCNP assay using EV‐stripped, EV‐concentrated, and unprocessed urine from three healthy donors. (g) Performance of the assay was also studied using EV‐ and PE‐derived from the BlCa and BPH urine pool, where the assay exhibits a higher S/B ratio for EV fractions compared to PE fractions. (h) The S/B ratio of uEVs measured by the CD63‐UCNP assay shows a significantly higher level of CD63‐positive EVs in BlCa patients compared to healthy individuals and patients with BPH. Here, the violin plots illustrate the distribution of S/B ratios of each group, with statistical significance reported by *p* values. NTA, nanoparticle tracking analysis; PE, protein‐enriched; S/B, signal‐to‐background ratio; TEM, transmission electron microscopy; TRF, time resolved fluorescence.

The limit of detection (LoD) of CD63‐UCNP‐LFIA was determined using DU145‐EVs spiked in EV‐stripped healthy urine matrix (EV‐removed urine). A serial dilution of EV particles, 1 × 10^7^, 2 × 10^7^, 4 × 10^7^, 8 × 10^7^, 1.6 × 10^8^, 3.2 × 10^8^, 6.4 × 10^8^, 1.3 × 10^9^ EVs/mL, was tested. The blank, 1 × 10^7^, 2 × 10^7^ and 4 × 10^7^ calibrators were analysed in 10 replicates. The calibrators 8 × 10^7^ and 1.6 × 10^8^ were analysed in six replicates. The calibrators 3.2 × 10^8^ and 6.4 × 10^8^ were analysed in four replicates. The calibrator 1.3 × 10^9^ was analysed in two replicates. LoD figure (Figure 2d) was determined by fitting the data to sigmoidal and logistic regression with Origin 2016 software. The LoD of the assay was calculated by multiplying 3 × SD (standard deviation of blank), and then the value was divided by the slope of the standard curve at the linear response.

### 2.10 Statistical Analysis

The LoD was calculated using Origin 2016 (version 2016Sr2). Statistical analysis was performed using R software (http://www.r‐project.org/), version 3.6.2. Box plots were done with Tidyverse (version 1.3.0) (Wickham, H. et al., Welcome to the tidyverse. *J. Open Source Softw*. 4, 1686, https://doi.org/10.21105/joss.01686 (2019)) and ggpubr (version 0.2.5) R packages (Kassambara, A. ggpubr: ‘ggplot2’ Based Publication Ready Plots. R package version 0.1. 7. Available from: https://CRAN.R‐project.org/package=ggpubr). For experiments consisting of more than two groups, we used the non‐parametric Kruskal–Wallis test where *p* value of <0.05 was considered significant.

## 3 Results

EVs from DU145 cancer cell line were isolated for assay development and standardization using UC. The quality of the isolated DU145‐EVs was assessed using various approaches, including analysis of particle size distribution by NTA, tetraspanin expression assessment by TRF immunoassays and morphological analysis through TEM. Figures 2a and 2b show a predominant particle size range and a high expression of tetraspanins CD63 and CD81 on DU145‐EVs. TEM imaging revealed the characteristic shape and structure of EVs with a lipid bilayer structure (Figure 2c).

The CD63‐UCNP‐LFIA is a lateral flow immunoassay designed to detect EVs via CD63 tetraspanin, using upconverting nanoparticles (UCNPs) to enhance sensitivity. The analytical performance of the assay was evaluated using purified DU145‐EVs as a standard. This assay demonstrated a LoD of 4 × 10^7^ EVs/mL (Figure 2d). To further evaluate potential assay interference and assess the linearity of the assay, serial dilutions of isolated DU145‐EVs were spiked in healthy pooled urine resulting in a response curve that shows raising average signals along with the increasing amount of spiked EVs (Figure 2e).

To evaluate the specificity of the assay, three types of urine samples were analysed as follows: unprocessed, EV‐stripped and EV‐concentrated urine. The EVs were stripped and concentrated from the urine of three healthy donors by ultrafiltration. The signals obtained with the different samples were then compared (Figure 2f). As expected, EV‐concentrated urine showed a 1.5–3‐fold higher S/B ratio compared to unprocessed urine and a 4–8‐fold higher S/B ratio compared to EV‐stripped urine.

Additionally, the performance of this assay was tested using EV‐ and protein‐enriched (PE) fraction derived from BlCa and BPH urine pools isolated by UC. In both BlCa and BPH, EV fraction exhibited almost a two‐fold higher S/B ratio compared to the PE fraction (Figure 2g).

The clinical performance of CD63‐UCNP‐LFIA was evaluated by quantifying CD63‐positive uEVs from healthy individuals (*n* = 30), patients with BPH (*n* = 50) and those with BlCa (*n* = 62). The assay demonstrated a significantly higher level of CD63‐positive EVs in BlCa patients compared to BPH (2.3‐fold, *p* = 0.007) and healthy controls (16‐fold, *p* = 0.00001) (Figure 2h).

## 4 Discussion

In this study, we demonstrate the utility of the UCNP‐LFIA for the detection of EVs from minimally pre‐processed urine, specifically targeting CD63‐positive EVs. The CD63‐UCNP‐LFIA employs anti‐CD63 antibodies for capturing and detecting EVs, enabling sensitive detection of CD63‐positive EVs in urine samples.

Several studies have previously explored the use of LFIA for EV detection, utilizing different nanoparticle label technologies, such as fluorescent nanospheres (Dong et al. 2019), colloidal gold nanoparticles (Yu et al. 2020; Oliveira‐Rodríguez et al. 2016) and Fe_3_O_4_ nanozymes (Wang et al. 2022), to enhance sensitivity. These studies report a wide range of LoDs, from 2.0 × 10^6^ to 1 × 10^10^ EVs/mL. However, direct comparisons of these LoD values are challenging due to the differences in standard materials and assay conditions across the studies. Additionally, many of these assays require labour‐intensive sample pre‐processing and isolation steps to achieve detection. While clinical validation of LFIA‐based EV assays has been limited, a recent study demonstrated the feasibility of using clinical samples in an LFIA platform (Lu et al. 2023). In contrast to other approaches, our LFIA‐UCNP provides the distinct advantages of detecting EVs directly from unprocessed urine, with an LoD of 4 × 10^7^ EVs/mL, offering a balance between high sensitivity and minimal sample preparation.

A previous study reported that the levels of CD63‐positive uEVs are significantly higher in bladder cancer patients compared to healthy controls (Liang et al. 2017). Our assay successfully detected CD63‐positive EVs in urine samples from healthy donors and patients with benign urological conditions and bladder cancer. Notably, no prior studies in the LFIA system have yet included any benign samples to distinguish them from malignant conditions. In our study, the inclusion of benign samples showed an overlap in S/B ratios between the bladder cancer and benign groups, suggesting commonality in uEV profiles and highlighting the complexity of relying on CD63 alone as a biomarker for cancer detection (Figure 2h).

The results of this proof‐of‐concept assay call for further clinical evaluation with more extensive and diverse patient material to clarify the clinical significance of CD63‐positive uEVs in the context of bladder cancer. In the future, the UCNP‐LFIA platform could be further developed for multiplexing to detect multiple biomarkers simultaneously, offering a more comprehensive readout. Our findings also show promise for the expansion of this assay concept further to detect other urogenital pathologies.

## 5 Conclusion

The developed CD63‐UCNP assay, where UCNPs were utilized in LF platform, serves as a straightforward method to identify CD63‐positive urinary EVs. This technology has the potential to be utilized in rapid, cost‐efficient and non‐invasive point‐of‐care applications. However, analysis of more clinical samples is imperative to confirm the effectiveness of this approach.

## Author Contributions

**Md. Khirul Islam**: conceptualization; data curation; formal analysis; investigation; methodology; validation; visualization; writing–original draft. **Imran Mahmud**: data curation, investigation, visualization, writing‐original draft. **Klinton Ali**: data curation; investigation; methodology; validation. **Teppo Salminen**: conceptualization; methodology; project administration; supervision; writing‐review and editing. **Pekka Taimen**: resources; writing–review and editing. **Peter J. Boström**: resources, writing‐review and editing. **Janne Leivo**: conceptualization; funding acquisition; project administration; resources; writing–original draft; writing–review and editing. **Urpo Lamminmäki**: funding acquisition; project administration; writing–review and editing. **Iida Martiskainen**: conceptualization, resources, supervision, writing‐original draft, writing‐review and editing

## Conflicts of Interest

The authors declare no conflicts of interest.

## Data Availability

The authors have nothing to report.
